# Infrainguinal inflow assessment and endovenous stent placement in iliofemoral post-thrombotic obstructions

**DOI:** 10.1186/s42155-018-0038-9

**Published:** 2018-11-16

**Authors:** Ole Grøtta, Tone Enden, Gunnar Sandbæk, Gard Filip Gjerdalen, Carl-Erik Slagsvold, Dag Bay, Nils-Einar Kløw, Antonio Rosales

**Affiliations:** 1 Division of Radiology and Nuclear Medicine, Oslo University Hospital, PO Box 4950, Nydalen, N-0424 Oslo, Norway; 2 Department of Vascular Surgery, Oslo University Hospital, PO Box 4950, Nydalen, N-0424 Oslo, Norway; 3 Section of Vascular Investigations, Department of Vascular Surgery, Oslo University Hospital Aker, PO Box 4950, Nydalen, N-0424 Oslo, Norway; 4 Institute of Clinical Medicine, Faculty of Medicine, University of Oslo, PO Box 1171, Blindern, 0318 Oslo, Norway

**Keywords:** Endovenous, Infrainguinal, Post-thrombotic, Inflow, Stent

## Abstract

**Purpose:**

To assess the technical success, patency, and clinical outcome, following assessment of inflow and infrainguinal endovenous stent placement in patients with iliofemoral post-thrombotic obstruction with infrainguinal involvement.

**Methods:**

A retrospective analysis of 39 patients with iliofemoral post-thrombotic venous obstruction accepted for infrainguinal stent placement in the period November 2009–December 2016. The clinical status was categorized according to the Clinical Etiological Anatomical Pathophysiological (CEAP) classification and symptom severity was assessed using Venous Clinical Severity Score (VCSS). The inflow was categorized as “good”, “fair”, or “poor” depending on vein caliber and extent of post-thrombotic changes in the inflow vessel(s). Stent patency was assessed by duplex ultrasound. Median follow-up was 44 months (range 2–90 months).

**Results:**

Stent placement was successful in all 39 patients. Primary patency after 24 months was 78%. Thirty of 39 patients (77%) had open stents at final follow-up. Re-interventions were performed in four patients and included catheter-directed thrombolysis (CDT) in all and adjunctive stenting in two. Twenty-eight of 39 patients (72%) reported a sustained clinical improvement. Patients with “good” inflow had better patency compared to those with “fair”/“poor” (*p* = 0.01). One patient experienced acute contralateral iliofemoral thrombosis; this segment was successfully treated with CDT and stenting. No other complications required intervention.

**Conclusion:**

Infrainguinal endovenous stent placement was a feasible and safe treatment with good patency and clinical results, and should be considered in patients with substantial symptoms from post-thrombotic obstructions with infrainguinal involvement. Stents with good inflow have better patency and inflow assessment is essential in deciding the optimal stent landing zone.

## Introduction

In chronic iliac vein obstructions percutaneous endovenous treatment with recanalization and stent placement, has shown to be effective for both post-thrombotic obstructions and non-thrombotic iliac vein lesions (Neglen et al., 2007; Hartung et al., 2009). However, when post-thrombotic obstruction extends below the inguinal ligament the role of endovenous stenting is not yet established (Neglen et al., 2008; Garg et al., 2011; Wittens et al., 2015). All treatment options for common femoral, deep femoral, and femoral vein obstructions aim at achieving long-term venous patency by optimisation of inflow and prevention of re-thrombosis. In addition to stenting, these options have involved open endophlebectomy, either solely or in combination with stenting and/or a temporary arteriovenous fistula (Vogel et al., 2012; Comerota et al., 2010). Involvement of the anatomical landmark of the sapheno-femoral junction has been used to guide the treatment option decision (van Vuuren et al., 2017a). However, the results following hybrid procedures have been disappointing with low primary patency and frequent complications and re-interventions (Garg et al., 2011; van Vuuren et al., 2017a, b, c; de Wolf et al., 2017). On the other hand, and as for arterial disease (Stricker and Jacomella, 2004; Rits et al., 2008; Goueffic et al., 2017) stent placement in the groin is rarely complicated with stent fracture (Neglen et al., 2008; Rosales et al., 2010).

The optimal endovenous stent placement is from one healthy vein segment to the next, obtaining adequate inflow and outflow (Neglen et al., 2008). In extensive post-thrombotic obstructions involving the infrainguinal veins, the optimal stent placement with regard to inflow is often challenged. In approximately 10–15% of patients with chronic venous femoropopliteal obstruction the deep femoral vein caliber and flow may increase over time, hereby transforming the deep femoral vein to a major outflow collateral. This transition of the deep femoral vein is termed axial transformation. Following axial transformation the deep femoral vein may serve as an alternative inflow path for stenting and a potential stent landing zone (Raju et al., 1998; Raju 2007).

Measures of venous pressure have shown to be unreliable and there is a lack of standardized and reliable methods to assess inflow (Rosales et al., 2010; Rosales and Sandbaek, 2013). As a consequence in clinical practice multimodality vein imaging including colour duplex ultrasound (CDU), intravascular ultrasound (IVUS), MR venography (MRV), CT venography (CTV), and conventional venography (CV), remains crucial in the diagnostic work up. We hypothesise that with adequate diagnostic imaging of infrainguinal inflow, endovenous stent placement in the groin can be effective also when involving the deep femoral and femoral vein.

The aim of this study was to assess the technical success, patency, and clinical outcome following infrainguinal endovenous stent placement, with assessment of inflow in patients with extensive iliofemoral post-thrombotic obstructions involving the common femoral, deep femoral, and femoral vein.

## Methods

The study was approved by the local Data Protection Official for Research, and by the Regional Committee for Medical and Health Research Ethics.

### Study design

This was a retrospective study of patients with chronic unilateral iliofemoral post-thrombotic obstruction with infrainguinal involvement, receiving endovenous stents extending below the inguinal ligament at a tertiary referral center from November 2009 to December 2016.

Treatment was offered to patients with active or recurrent ulcer, venous claudication, edema, or severe pain. Patients with bilateral post-thrombotic obstructions, or with involvement of the inferior vena cava, were not included. Patients with obstructions as a result of malignancy or drug abuse were not included. The patients’ pre- and postoperative hospital medical records were obtained including CDU, MRV, CTV, and CV, and full medical history following the referral. Follow-up visits were performed at 3, 6, 12, 18, and 24 months, and yearly thereafter and included clinical evaluation and CDU. Stent patency was defined as antegrade flow throughout the stented segment on CDU, either spontaneous with respiration dependent flow or with distal pneumatic compression in order to provoke flow. In case of a non-conclusive CDU examination a supplementary CTV was performed to confirm stent patency or occlusion. At each visit current symptoms of chronic venous disease (CVD) including edema, heaviness, venous claudication, and/or ulcers were registered. Patients’ clinical status was categorised according to the C component of CEAP (Clinical Etiological Anatomical Pathophysiological) classification, and symptom severity was assessed using the venous clinical severity score (VCSS).

### Baseline imaging and assessment of inflow

**Fig. 1:**
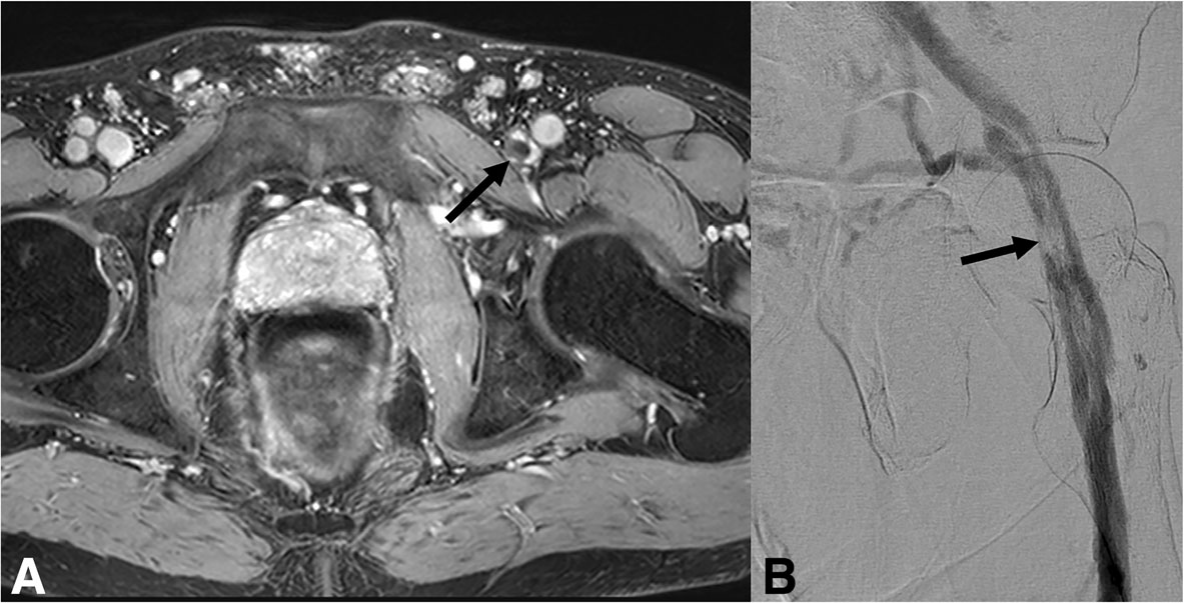
Imaging of typical post-thrombotic obstruction (black arrows) of the left common femoral vein at the level of the femoral head on MRV (**a**) and CV (**b**), respectively

Initially, there was no pre-defined work-up for diagnostic imaging after CDU and ambulatory venous pressure measurement. CTV was performed in 20 patients. CV with popliteal access was performed in 12 patients, and seven patients had CV with access in a superficial vein on dorsum of the foot. In 2013 MRV (Enden et al., 2010) was introduced as routine imaging; of the 12 patients examined with MRV three had an additional CV from the popliteal vein. In nine patients MRV was the only imaging modality before the recanalization procedure (Fig. 1).

**Fig. 2:**
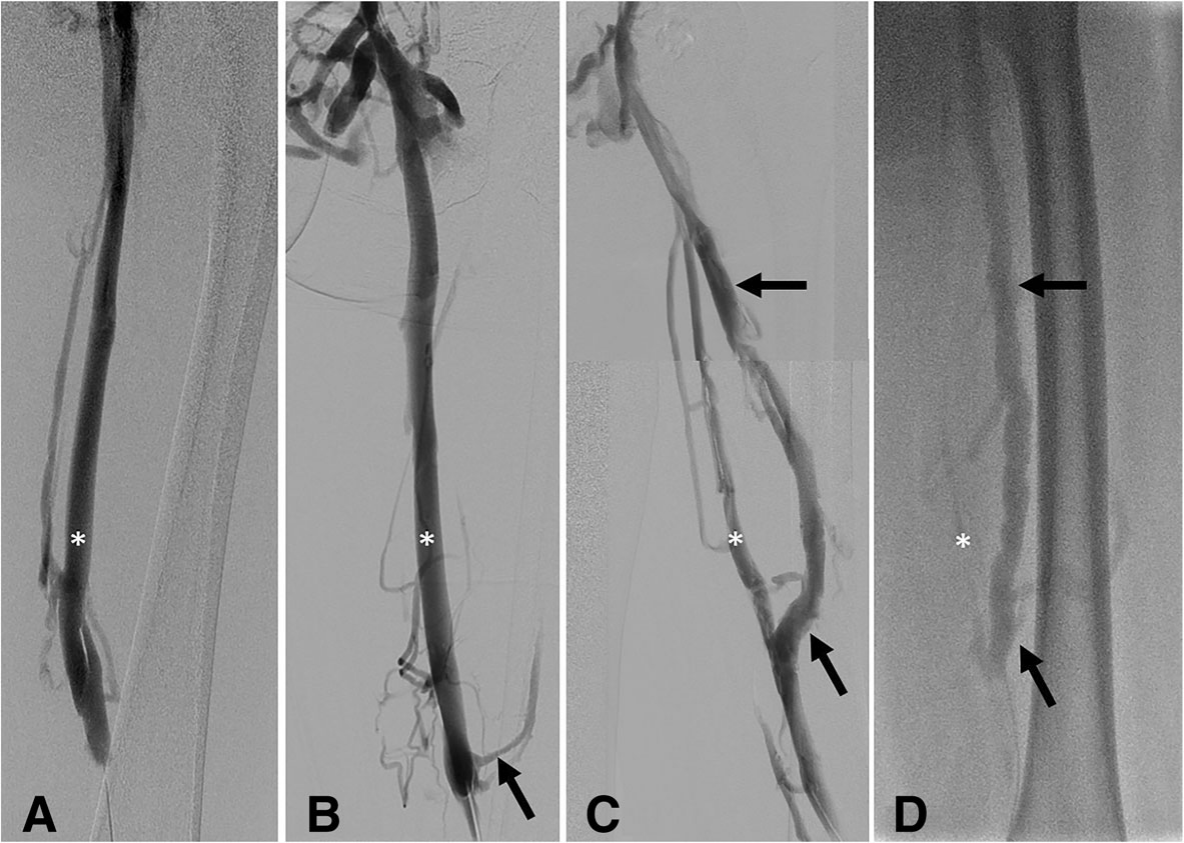
Examples of grading of axial transformation of the deep femoral vein on conventional venography. **a** Grade 0, normal anatomy with no post-thrombotic changes. **b** Grade 1, a popliteal-deep femoral collateral connection (black arrow) and normal caliber deep femoral vein. **c** Grade 3, the deep femoral vein (black arrows) is larger than the obstructed femoral vein. **d** Grade 4, large caliber deep femoral vein (black arrows) and occluded femoral vein. White * indicates the femoral vein

**Fig. 3:**
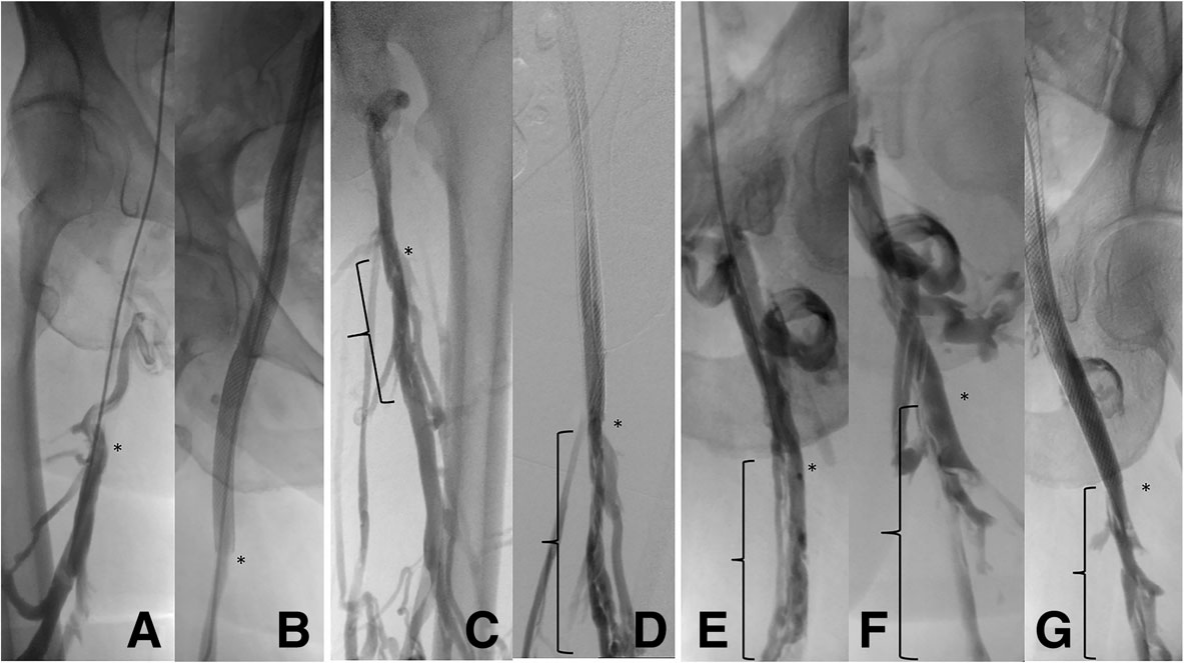
Examples of classification of deep femoral vein inflow.^0^ **a**, **b** Good inflow with a grade 3 axial transformation. **c**, **d** Fair inflow with a grade 3 axial transformation and post-thrombotic obstruction caudal to lower stent border. ^0^ **e**, **f**, **g** Poor inflow with grade 2 axial transformation and post-thrombotic obstruction of both the femoral and deep femoral vein caudal to lower stent border. ^0^ **a**, **b** and **e**, **f**, **g** With contrast injection into the deep femoral vein and no opacification of the femoral vein. * indicates the level of the lower stent border in the deep femoral vein. {indicates post-thrombotic segment caudal to lower stent border

For a systematic, descriptive assessment and classification of inflow pre- and per-procedural vein imaging were reviewed by the principal investigator who at that time was unaware of the long-term post-procedural outcomes. The pre-stent assessment involved presence of post-thrombotic changes in and below the segment undergoing treatment and the overall caliber of inflow vessels. Axial transformation of the deep femoral vein was classified according to Raju (Raju et al., 1998) (Fig. 2). The inflow was classified as “poor” in the presence of small caliber inflow vessels with post-thrombotic obstructions. “Good” indicated no or discrete post-thrombotic changes in adequate caliber inflow vessel(s), hereunder axial transformation of the deep femoral vein grades 3 and 4. The cases that did not fit into inflow categories “poor” or “good” were classified as “fair” (Fig. 3a-g). In patients with a poor inflow to the common femoral vein, the lower stent border was placed into the deep femoral or femoral vein.

### Endovascular procedure

The endovascular procedure was performed in a hybrid suite under general anaesthesia and full anticoagulation pre-, per- and postoperatively with low molecular weight heparin; dalteparin 100 IE/kg twice daily. Ultrasound guidance was used to gain vein access; for the great majority (*n* = 34) through the right internal jugular vein. Three patients received an additional access in the common femoral vein because of failed recanalization from above. One patient had an additional access from the popliteal vein for assessment of inflow. In the remaining five patients four had access through the popliteal vein only, and one in the common femoral vein. The obstructed vein segments were passed using stiff hydrophilic 0.35 or 0.18 guidewires supported by a Cobra C2 catheter (5 French or 4 French hydrophilic). Following CV with assessment of inflow and stent landing zone a non-hydrophilic supportive guidewire (Amplatz Super Stiff; Boston Scientific Corporation, Natick, MA, USA) was introduced. A gentle balloon inflation just distal to the planned level of lower stent border to exclude stenosis not revealed on CV was performed before predilatation throughout the obstruction with large size balloons (Atlas; Bard, Covington, GA, USA) sized equal or 2 mm less than stent diameter. Self-expanding braided stainless steel stents (Wallstent; Boston Scientific Corporation, Natick, MA, USA) were placed with overlap. When possible, the infrainguinal stent was placed first followed by the most cranial stent. Both stents were post-dilated before the segment in between was stented and aiming at stent overlap of 2 cm. Iliac stent diameter was 14–18 mm and infrainguinal stent diameter was 12–14 mm. Intermittent pneumatic compression was applied immediately following the procedure and continued while the patient was constrained to bed. The patients received dalteparin for at least 3 months followed by transition to oral anticoagulation with warfarin (international normalized ratio 2.5–3.5) during follow-up. Yearly, continued anticoagulation was discussed with the hematologist.

### Statistics

Data were analysed with SPSS version 24 (IBM, Armonk, NY, USA). A Wilcoxon signed rank test was used for evaluation of difference between clinical scores at baseline and at final follow-up. Primary and secondary stent patency were evaluated with Kaplan-Meier analysis. A log-rank test was used to compare patency rates. Results with a *p*-value < 0.05 were considered statistically significant.

## Results

### Patient characteristics

Thirty-nine patients were included, see Table 1 for baseline patient characteristics. Thirty-three patients were screened for thrombophilia; 21 of these with positive findings hereunder 14 with a severe thrombophilia (six with protein S deficiency, two with protein C deficiency, three with homozygous factor V Leiden polymorphism, two with antithrombin deficiency, and one with a combination of prothrombin and heterozygous factor V Leiden polymorphisms). Median time from thrombotic event to stent placement was 142 months (range 19–468 months).

**Table 1:** Baseline patient characteristics

Age, years (median – range)			46 (26–74)
Female, n (%)			26 (67)
Left limb involvement, n (%)			32 (82)
Venous claudication, n (%)			34 (87)
VCSS, mean (range)			8 (2–20)
CEAP	C0		2
	C3		22
	C4		3
	C5		8
	C6		4

All 39 patients presented with CVD symptoms of the lower limb. In the categories C0-C4 venous claudication was the indication for treatment in 25 of 27 patients. The indication for treatment in the final two patients was severe edema assumed to contribute to erysipelas and septicaemia, and large suprapubic varicosities, respectively. Twenty-five patients had May-Thurner diagnosed on either CTV or MRV. A summary of axial transformation is presented in Table 2.

**Table 2:** Axial transformation of the deep femoral vein and lower stent border

Axial transformation^a^	Lower stent border		
	Common femoral vein^b^	Deep femoral vein	Femoral vein
Grade 0	5	0	1
Grade 1	4	0	0
Grade 2	2	3	0
Grade 3	10	9	0
Grade 4	1	2	0

^a^Grade 0 = normal vein anatomy, grade 1 = popliteal-deep femoral vein collateral connection with normal deep femoral vein caliber, grade 2 = enlarged deep femoral vein smaller than or equal to the femoral vein, grade 3 = deep femoral vein larger than obstructed femoral vein, grade 4 = total occlusion of femoral vein^14^

^b^Missing data *n* = 2

### Technical success and procedural details

Recanalization and stent placement were technically successful in all 39 patients. Twenty-four patients had stents extending into the common femoral vein, 14 had stents extending into the deep femoral vein and one patient had stent extending into the femoral vein. For summarising results please see Table 3. One patient received one stent, 15 patients received two stents, 21 patients received three stents, one patient received four stents, and one patient received five stent. Cranially all stents extended into the confluence of the inferior vena cava except in one patient where the cranial stent was placed in the external iliac vein due to a normal common iliac vein.

**Table 3:** Infrainguinal stent placement; baseline inflow, clinical results, and patency at final follow-up

	Baseline inflow			Final follow-up					
				VCSS			Patency		Complete recovery from venous claudication
	Good	Fair	Poor	Decreased	Unchanged	Increased	Yes	No	
All patients (*n* = 39)	24	13	2	28	8	3	30	9	23 of 34
Lower stent border in the									
Common femoral vein (*n* = 24)	18	5	1	16	6	2	21	3	14 of 19
Deep femoral vein (*n* = 14)	5	8	1	11	2	1	8	6	8 of 14
Femoral vein (*n* = 1)	1	0	0	1	0	0	1	0	1 of 1

*VCSS* venous clinical severity score

### Clinical effects

Twenty-eight of 39 (72%) patients reported clinical improvement including healing of ulcer in two of the four C6-patients. Twenty-three of 34 (65%) patients with venous claudication at baseline reported no such symptoms at final follow-up. Median follow-up was 44 months (range 2–90 months). At final follow-up the mean VCSS score was 6 (range 0–20) and VCSS decreased in 28 patients (*p* < 0.001). Three patients with unchanged CVD symptoms had a higher VCSS score at final follow-up compared to baseline because of compression therapy initiated after stent placement. Changes in VCSS and venous claudication with regard to baseline inflow and lower stent border are summarized in Table 3.

### Patency and re-interventions

At final follow-up 30 of 39 (77%) patients had patent stents. Seven of nine stent occlusions occurred before 18 months follow-up.

**Fig. 4:**
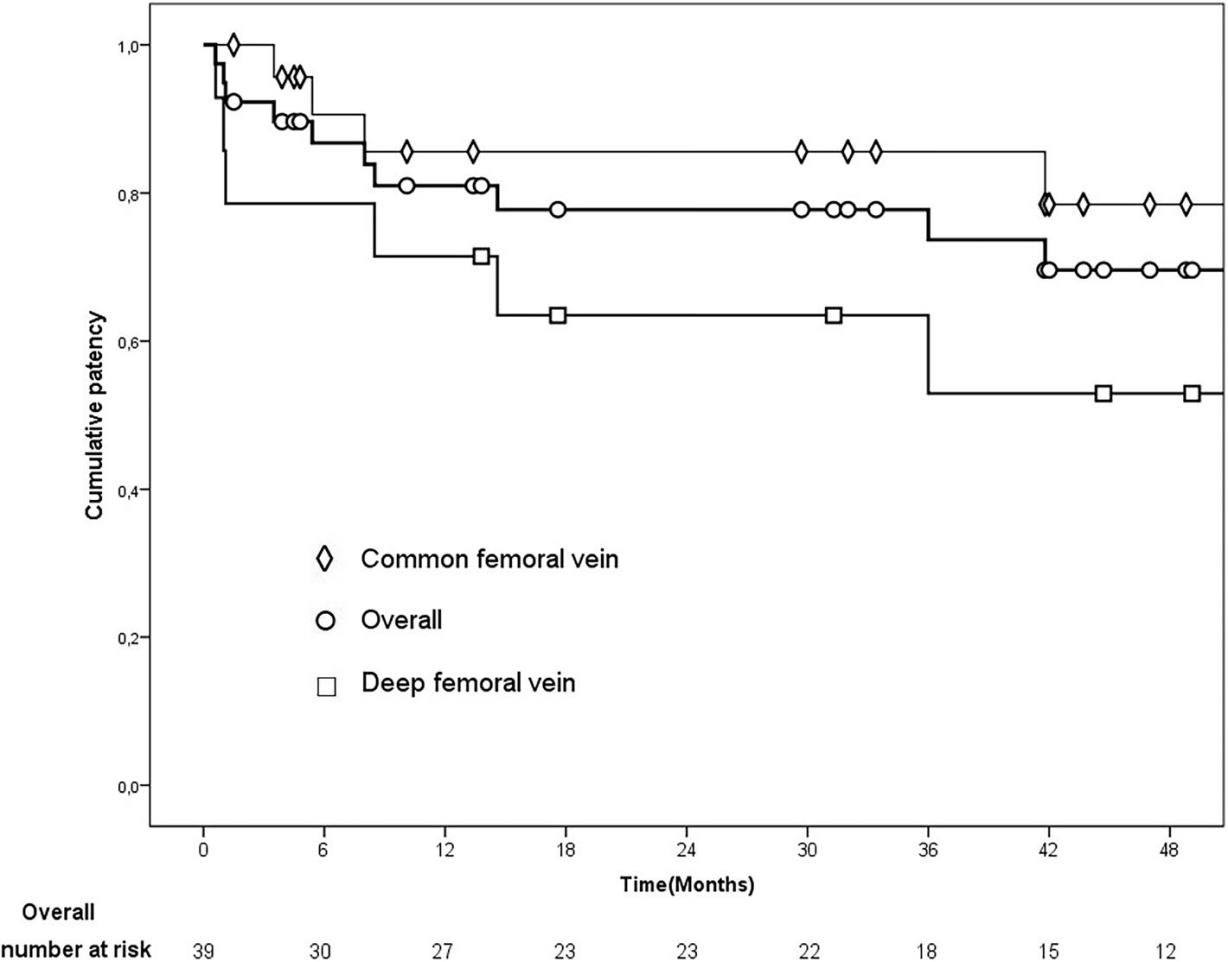
Cumulative primary patency for stents extending into the common femoral vein (◊), overall (**○**), and deep femoral vein (□),respectively

Following 24 months follow-up the overall cumulative primary patency was 78%. Correspondingly, 24 months patency was 86% for stents extending into the common femoral vein and 64% for stents in the deep femoral vein (*p* = 0.05) (Fig. 4). The estimated mean overall primary patency was 67.3 months (95% CI 55.1–79.4 months).

**Fig. 5:**
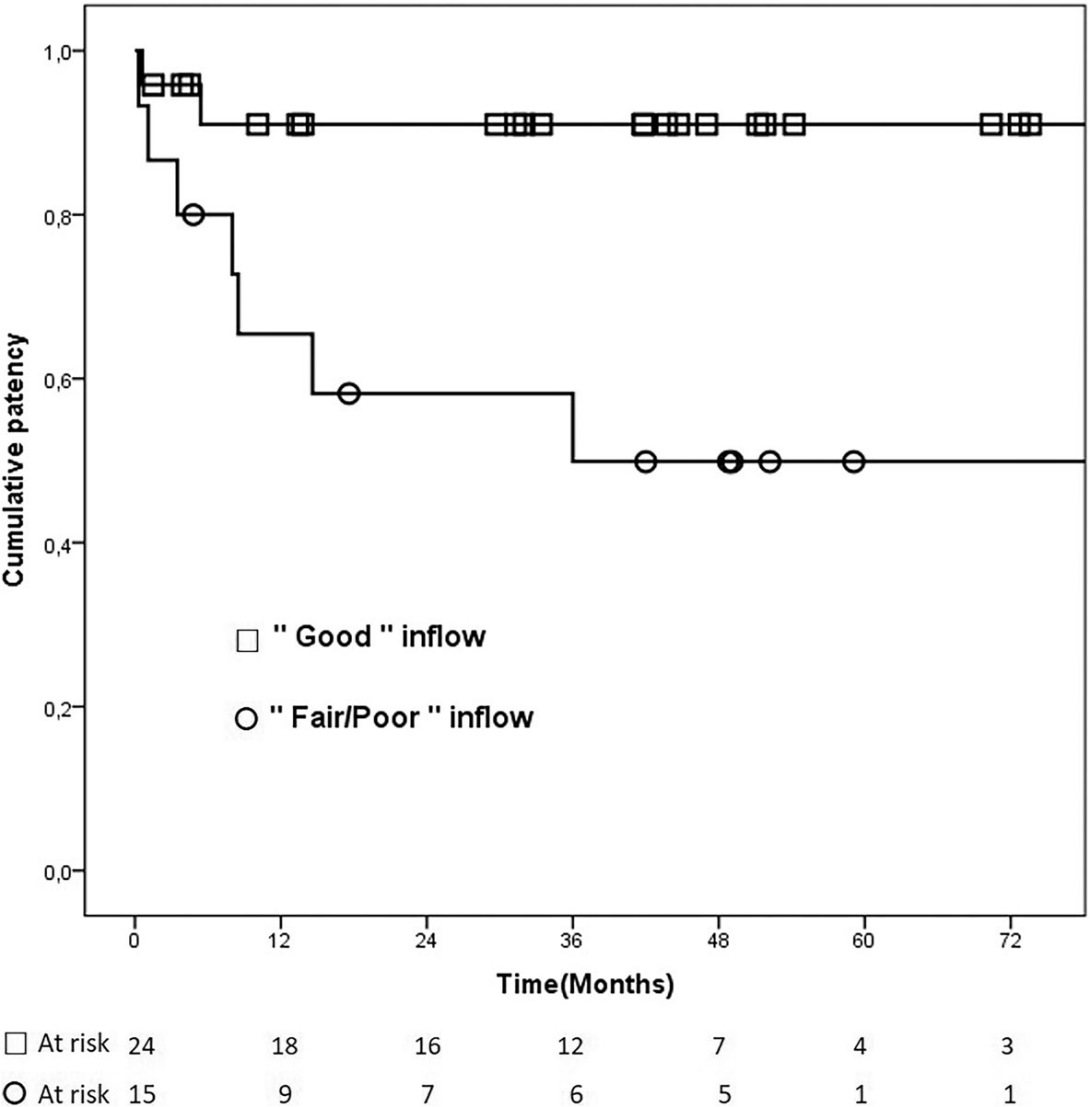
Cumulative primary patency in patients classified with “good” inflow (□) versus “fair/poor” inflow (○) (*p* = 0.01)

Both patients classified with “poor” inflow had occluded stents at final follow-up. Patients with “good” inflow had better 24 months patency compared to those with “fair” / “poor”, 91% versus 58%, respectively (*p* = 0.01) (Fig. 5).

Re-intervention was performed in four patients who presented with acute symptoms of stent occlusion. The primary and secondary patency in these patients were 17 days, one and two months, five and 10 months, and 42 and 51 months, respectively. One patient with “good” inflow in an axially transformed deep femoral vein grade 3 and cranial stent border in the inferior vena cava, experienced bilateral iliofemoral occlusion 17 days following stenting. This patient underwent re-intervention with catheter-directed thrombolysis (CDT) and stent placement in the contralateral iliofemoral segment which has remained open, but CDT failed on the ipsilateral side and no secondary patency was obtained. The second patient in the re-intervention group with stent extending into the deep femoral vein occluded after 1 month and was treated with CDT, but re-occluded 6 weeks after the re-intervention. Including this patient, three patients with occluded stent in the deep femoral vein reported cessation of intermittent claudication and no further re-intervention was performed. Two of the patients in the re-intervention group had stents extending into the common femoral vein and experienced stent thrombosis following cessation of anticoagulation. Both were treated with CDT; at final follow-up one had stent occlusion while the one who also had been re-stented because of residual thrombus had patent stents.

The remaining patients with stent occlusion at final follow-up received no re-intervention because they either presented with no associated symptoms or made contact or were referred too late for CDT. One patient reported post-procedural local numbness near the access site in the neck which was treated conservatively. There were no procedure related complications requiring intervention, hereunder no stent fractures.

## Discussion

In this retrospective study of 39 patients with iliofemoral post-thrombotic obstruction with infrainguinal involvement, endovenous infrainguinal stent placement was successful in all patients. The majority of patients reported clinical improvement and had patent stents at final follow-up. None of the patients with occluded stents reported clinical deterioration. Patients classified as having “good” inflow had better patency compared to those with “fair/poor”, and inflow seemed to be a more important predictor of patency than localization of lower stent border.

The two-year cumulative primary patency in our study was 78%. This is in line with the results reported by Neglen et al. on 53 patients with stents extending into the common femoral vein (Neglen et al., 2008). Van Vuuren et al. recently reported on patency and clinical outcome in 369 patients who received percutaneous stent placement or a hybrid procedure (combining stenting with open surgical disobliteration and arteriovenous fistula) depending on the extent of the infrainguinal post-thrombotic obstruction with regard to the sapheno-femoral junction (van Vuuren et al., 2017a, b, c). In this material the 60 months primary, primary assisted, and secondary patency in the stent group were 64%, 81% and 89%, respectively. However, 29% received re-intervention(s), which is higher than in our study. The corresponding patency rates in the hybrid group after 36 months follow-up were 37%, 62% and 72%, the complication rate was high with 89%, and 59% received various re-interventions (van Vuuren et al., 2017a, b, c). Poor results for the hybrid approach has also been reported in a small study by Garg et al. with a 24 months cumulative secondary patency of 30% (Garg et al., 2011).

Another recent report by the same Dutch group included 24 patients undergoing a scheduled procedure for closure of arteriovenous fistula following a hybrid procedure with stenting below the sapheno-femoral junction (van Vuuren et al., 2017a, b, c). The lower stent border was placed into the vessel with highest quality and flow as assessed on ascending venography. The primary, primary assisted, and secondary patency were 60%, 70% and 70%, respectively. In line with the findings in our study, the authors indicate that in patients with infrainguinal obstruction and sufficient inflow on imaging, primary stenting may be a favorable alternative to the hybrid approach.

Although the evidence of infrainguinal stent placement into the common femoral vein is growing, the experience with stent placement into the deep femoral and femoral vein is scarce (Wittens et al., 2015; Rosales et al., 2010; van Vuuren et al., 2017a, b, c). Moreover, given the low primary patency rate and high rates of re-intervention and complications following hybrid procedures, further research and development should aim at optimizing the solely percutaneous stenting technique. This also includes studying the efficacy of the new generation dedicated venous stents in infrainguinal venous obstructions. These nitinol stents have the advantage of easier deployment and various combinations of flexibility and radial force, with the potential of better performance compared to braided stainless steel stents.

The overall favorable outcomes following recanalization and infrainguinal stenting, and the few other treatment options available, justify a minimally invasive approach in these patients (Wittens et al., 2015). Moreover, the endovascular approach is safe and even with failed stent patency no clinical deterioration was reported in our study.

In spite of the dedicated follow-up and explicit patient information about the importance of immediate contact in the case of symptom recurrence, the majority of stent occlusions were detected at a planned follow-up visit. This may have contributed to the low number of re-interventions in this study. Accordingly, the quality of the patient information may be improved, however the delay in diagnostics may also be explained by insidious development of in-stent restenosis with little symptoms, or a limited immediate access to health care.

In our study the stented segments in the two patients who were classified as having “poor” inflow occluded after 8 months. Hence, endovenous stent placement may not be justified for small caliber inflow vessels with post-thrombotic changes, and a hybrid approach can be an alternative treatment approach in these patients (van Vuuren et al., 2017a, b, c).

Two of the 24 patients classified as having “good” inflow experienced stent occlusion. One occluded following cessation of anticoagulation. The other patient experienced early stent occlusion with concurrent contralateral iliofemoral occlusion; a rare, but previously reported complication for stent protrusion into the inferior vena cava (Neglen et al.,^2007^). The etiology of the bilateral occlusion in this patient with no known thrombophilia was not identified, and other factors than inflow may have contributed to the stent occlusion.

Limitations to this study include its retrospective design, the low number of patients and no control group. Although IVUS is becoming an established image modality for venous assessment, IVUS was not used in our study. However, it has been reported that IVUS has higher sensitivity for the detection of venous stenosis compared to CV (Neglen and Raju, 2002; Gagne et al., 2017). Post-thrombotic changes detected on IVUS and not on CV, may improve the assessment of stent landing zone (Neglen and Raju, 2002). The Villalta scale has been recommended for the diagnosis and assessment of post-thrombotic syndrome (PTS) in clinical trials (Kahn et al., 2009). With our retrospective study design it was not possible to provide Villalta scores. However, good correlation between Villalta scale and VCSS has been shown, and VCSS may be more sensitive in severe PTS (Jayaraj and Meissner, 2014).

Our findings confirm the importance of adequate venous inflow, and indicate that the suggested inflow classification may be valuable in guiding the decision on where to place the lower stent border, and possibly which patients may not be suitable for endovascular treatment. However, the validity and reliability of such a classification needs to be applied and assessed in a larger patient cohort. Nevertheless, we think that in addition to MRV patients with post-thrombotic obstruction in the femoral confluence, should be examined with a CV with contrast injection from the dorsum of the foot, or from the popliteal vein, to evaluate axial transformation and inflow to improve the decision on optimal stent landing zone.

Although there are reports on the benefit of endovenous stenting, controlled trials have been lacking. A randomised study comparing venous stenting with conservative treatment is ongoing and its research protocol has been published (van Vuuren et al., 2017a, b, c).

## Conclusion

Infrainguinal endovenous stent placement was a feasible and safe treatment with good patency and clinical results, and should be considered in patients with substantial symptoms from post-thrombotic obstructions with infrainguinal involvement. Stents with good inflow have better patency and inflow assessment is essential in deciding the optimal stent landing zone.

## Abbreviations

CDT: Catheter directed thrombolysis
CDU: Colour duplex ultrasound
CEAP: Clinical etiological anatomical patophysiological
CTV: Computer tomography venography
CV: Conventional venography
CVD: Chronic venous disease
IVUS: Intra vascular ultra sound
MRV: Magnetic resonance venography
VCSS: Venous clinical severity score
